# Medicine in 1950—A Look Forward

**Published:** 1912-03

**Authors:** W. T. Marrs

**Affiliations:** Peoria, Ill., Author of “Confessions of a Neurasthenic”


For Texas Medical Journal.

### Medicine in 1950—A Look Forward.

BY W. T. MARRS, PEORIA, ILL.,
Author of "Confessions of a Neurasthenic."

A good many men and women now entering upon the study and practice of medicine will still survive in 1950, which is a little less than forty years in the future. It is hardly a wise bit of speculation to say that the practice of medicine at the close of four more decades will have been revolutionized, for human anatomy and physiology do not in the different ages admit of only about a certain line of lesions and pathological changes. Still medicine and surgery *as practiced* will be quite different from its status now.

Just a moment of retrospect first. During the past forty years we have been going considerable. Along about 1880, which time many of us can let memory rather distinctly slide back, things medical were quite different from what they are now. About our best diagnostic agents then, in addition to what nature gave us, were the clinical thermometer, the stethoscope, the crude urinary tests, and a few others. The only specific agency at that time was vaccination as a preventive of smallpox. We have now a few specifics and several near-specifics, with vaccine therapy and specific medication just entering its lusty infancy. Our diagnostic paraphernalia and our acumen to use them are wonderful as compared with forty years ago, and it is only natural to assume that we will make progress along several lines of endeavor during the four decades to come.

In 1950 a great many diseases and disease terms now found in our text-books will have become obsolete and will be gradually lopped off. In passing, I wish also to state that many of our awkward words and lumbering terms used only in medical writings will be relegated to the unknown whence. We shall not speak of "exhibiting" a drug and "purifying the blood" and "eliminating toxines." Our terms and nomenclature will be clear, and we shall describe a thing tersely and directly, just as a newspaper man now tells his story.

Among the many diseases that will be *passe* can be mentioned typhoid fever. Several thousands die annually in the United States of this disease. It is a disease of filth and bad sanitation, and these conditions will be done away with and typhoid fever will go out. Recently it was observed in Texas what a benign action was played by the use of typhoid vaccine, as no cases of this disease occurred among the soldiers encamped there. Malaria will have disappeared in less than forty years among all people who have good drainage and effective sanitary conditions. The mosquito with his poisonous malarial proboscis will soon be exterminated. A number of infectious diseases will become in a measure extinct and when they do occur will respond to a specific.

Very few powerful remedies will be employed. In fact, not many of any kind of drugs will be in use as compared with the thousands of drug agencies and combinations now employed. Our present materia medica will amuse students; it will be expurged of hundreds of its useless or unnecessary drugs. The materia medica will be cleaner, better and more easily mastered when we have thrown out a lot of our old weeds and rubbish. Poison drugs will be seldom dispensed; i. e., strongly toxic agencies. Bichloride of mercury and carbolic acid will have given way to less powerful germicidals. Cyanide of potassium and a number of other useless agencies—only for self-extermination—will be suppressed by law.

Remedies will not go out of style, but they will be much more intelligently used than now. If, for example, it is thought that a patient needs "beef, wine and iron," we would not get it from the shelves of a drug store but each agency will be used separately and in the best form. The proprietary medicine business will have taken quite a slump and only the dependable ones will be exploited, for it is for the most part a wise and discriminating public to which they appeal. There are perhaps a dozen preparations now advertised in medical journals that will be before the people in that day. It would be unfair for the self-appointed seer to mention any names.

Tuberculosis will not have been wiped out, but it will be 75 per cent less common and fatal than now. Clean, wholesome living and the avoidance of killing occupations will help to lessen the disease. The tenements of the large cities will no longer be so crowded and consequently the diseases of squalor and poverty will abate. Good living and good nutrition will in a measure solve the tuberculosis question. Even now—1912—it is claimed that fully half the persons who become infected will and do recover under proper feeding and care. The searchlight of knowledge is doing much in this tuberculosis matter. In a few years it will be considerably suppressed, and in consequence our progeny will grow stronger and better capable of coping with the mighty tubercle bacillus. But consumption we will have for many, many decades to come, for it is hardly to be supposed that people universally will ever rigidly obey the laws of health and hygiene.

As fevers and acute and infectious diseases become less and less, those of a neurotic trend will become more numerous. It will be an age of wits, of education, of sharp competition. Few will care to do rough work and most everybody will want to direct his work through a system. It will be a time and age that will draw heavily on our gray matter and our neurons. Mental ailments, neuroses and psychoses will be abundant, although symptoms will be named and classified differently than now. Such terms as "hysteria," "neurasthenia," "psychasthenia anorexia," and many others will be obsolete.

The sanitariums and hospitals will nearly all be for people suffering from broken and decrepit nerves and the ailments incident to old age. It will be the endeavor of psychiatrists to cure people before they become insane instead of after. The insanity of the future will be of mild type and we will look on such sufferers as having sick nerves or sick brains, just as we now regard a sick and inefficient stomach or a broken limb. In fact, the greater number of diseases will originate from within and not from without. The denizens of that day will have little fears from germs but most of their troubles will be due to toxines manufactured within the body and, greater still, to the wear-and-tear and disintegration of cells and tissues. Still people will live longer then than now. Longevity is constantly increasing, and in forty years the average length of life will be extended ten years.

Psychiatry has been casually mentioned. We knew little about it twenty years ago—we know *a little* about it now and are learning more all the time. We will soon know something about cerebral localization, whereas we now see through a glass darkly. We shall some day cure many cases of epilepsy by surgery, in the removal of pressure from ganglionic centers. I have seen surgeons attempt this and fail; they didn't know what they were doing; they were toiling on an unknown sea without chart and compass. But some day we shall understand these things. Telepathy will be worked out; you and I may not live to see it, and it is hard for us to believe it. We thought that of the telephone and wireless telegraphy, though. The mind will be the great poaching ground in the future. We will better understand the brain and will earn to make use of the many unenergized talents that we now possess.

In 1950 the State will care for all the physically defective in suitable homes, but there will then be not so many defectives of certain kinds as now exist. For instance, congenital blindness or blindness from infancy will almost be unheard of. Most of the blindness being due to ophthalmia neonatorum or blenorrhagic disease, these matters will be more rigidly dealt with. Gonorrhea will be regarded with more seriousness than it once was and with its passing blindness will decrease and there will not be so many people with sightless orbs to care for. Neither will there be so many deaf, idiotic, epileptic, degenerate and otherwise morally, mentally and physically defective. This salutary state of affairs will be brought about not through any "serum" or specific, but through a more thorough knowledge of social matters and applied eugenics. Criminals and perverts will by law be rendered unable to procreate. A good many who suffer from transmissible diseases will feel it incumbent upon themselves to submit to sterilization in order that they may not bring into the world progeny that is physically and mentally handicapped. Many persons will submit themselves voluntarily to doctors for sterilization, but the profession will exercise a good deal of discretion and scrutiny in these cases, lest it be a mere pretext. The candidate may want to shirk paternity or he may want to become a social libertine. The doctors will recall the fact that forty or fifty years previously women had a penchant for becoming unsexed in order that they might escape maternity. But with a certain amount of care and restriction the profession in 1950 would not think of going back to the old order of things where every unfit individual of either sex was allowed to procreate at will—under the name of the law usually. There were, of course, many objectors to the sterilization of the unfit on sentimental grounds but that was finally overcome by hard heads and common sense. There is no sentiment, no charity, in letting people be born who would be better off if they had never been conceived.

There will not be so many divorces at that future date. It will not be difficult for the mismated and people of "incompatibility of temperament"—whatever that is—to become legally separated. But the courts will seldom be resorted to for such dissolution. Why? It will be more difficult to get married. Before being granted a license the candidates must convince a "fair and impartial" and *qualified* board that they are in every way fitted to enter the connubial state. Of course, there will be mistakes occasionally but no comparison to the old days when there was no restriction on the marriage ban. So it is nothing to marvel over that the people of the future, and not so distant future either, will be different from us in many ways. This improvement in race naturally changes the practice of medicine in some degree. The State will employ a great many physicians, and there will be much scrambling for the plums; there will also be some graft and crookedness, for human nature is as much a fixture as the rock of Gibraltar.

There will not be so many babies born as now, although the race will not be in any danger of dropping into desuetude. The babies, with few exceptions, will all live. It is as natural for a well-born and well-nurtured babe to live and grow as it is for any sort of vegetation to live and grow. It will be a good thing, this limiting of the number of offspring among the poor. On the other hand, doctors will have more practice among the rich and favorably environed, for even the disgustingly rich will then have more children than they do now. Educated women will not shirk maternity as they do at present; it being the natural heritage of the sex, they will look on motherhood from another—really the old—angle. Society will be considerably over its heydey, and, as every possible kind of thrill and excitement will have become old and exhausted, maternity then will get its deserts. Society and culture will be regarded in a different light from the way we now view these things. Everything will be practical and utilitarian; theoretical matters will have little weight in the profession or out of it.

Surgery will not be so radically different from now, only there will not be quite so much of it. Surgical skill has reached a state of technique now—1912—that is well-nigh marvelous. We have long had surgeons without number who, in the language of the unsophisticated layman, "can cut a man to pieces and put him together again." It would be difficult to conceive of how surgical skill could be very much improved when now we suture up heart lesions, make artificial anastomoses of blood-vessels, cut out diseased sections of intestines, extirpate the stomach (and let the patient live a while), correct atrocious hernias, make a homely individual handsome by cosmetic surgery, and countless other things.

Gynecological surgery will not be common. About 80 per cent of the pelvic cysts, suppurating ovaries and rotten pus tubes which make operative interference imperative will not occur very often, because the cause—the gonococcus—will not be so much in evidence. When these surgical conditions do arise in women the appendix may be involved and thus need removal. Suppurating appendices will occasionally occur in broken-down or tuberculous individuals, but rarely. The surgeons of 1950 in private conclave will sometimes say to one another: "Those old fellows back forty years ago surely had a snap. They could take out an appendix and make the fellow whack up a stiff fee with less circumspection than we can now lance a furuncle. There wasn't any officious medico-legal inspector then to butt in to see if everything was on the level. But it may be that the old surgeons worked the game a little too hard and thus caused the operation to fall into disrepute." Surgery will be conservative and no major operation will be allowed by law to be performed until certain requirements of a medico-legal character are complied with. Many will complain of these restrictions and call it legal red tape, but it will be a good thing for the public.

There will be fewer doctors than now, although there will be the usual complaint about a "crowded profession." The people will be more scattered then than at present, and so naturally the doctors will be too. The cities having grown so large and the manufacturing business having been so thoroughly overdone, more people have turned back to the soil trying to be producers. The more intelligent people will live in the country or suburbs and the best doctors follow civilization and culture as a patriot follows the flag. In the large cities there will always be doctors' offices "down town," but not nearly so many as now. Most of them will operate their business from one place. Thousands of doctors will at their residences have a diminutive sanitarium or hospital. Accidents which injure people will be fewer; the law will forbid the factories and railroads killing people. Much prescribing will be done without visiting the patient personally, for the instrument which is now our telephone will be so perfected that it will enable the physician to see and examine a person almost as satisfactorily as if he were in the same room.

The aeroplane will be very common and people will sail through the air with bird-like ease at the height of twenty to forty feet, but they will not be much used by doctors only for seeing patients at a long distance. The aeroplane is more cumbersome to use than the automobile. The autos will be run pretty much altogether by electricity, thanks to the new and inexhaustible storage battery brought out by Mr. Edison's successor.

The only way you can successfully contradict these prophecies is to be alive at that time and see for yourself.

